# The ASCC2 CUE domain in the ALKBH3–ASCC DNA repair complex recognizes adjacent ubiquitins in K63-linked polyubiquitin

**DOI:** 10.1016/j.jbc.2021.101545

**Published:** 2021-12-28

**Authors:** Patrick M. Lombardi, Sara Haile, Timur Rusanov, Rebecca Rodell, Rita Anoh, Julia G. Baer, Kate A. Burke, Lauren N. Gray, Abigail R. Hacker, Kayla R. Kebreau, Christine K. Ngandu, Hannah A. Orland, Emmanuella Osei-Asante, Dhane P. Schmelyun, Devin E. Shorb, Shaheer H. Syed, Julianna M. Veilleux, Ananya Majumdar, Nima Mosammaparast, Cynthia Wolberger

**Affiliations:** 1 Department of Biophysics and Biophysical Chemistry, The Johns Hopkins University School of Medicine, Baltimore, Maryland, USA; 2 Department of Science, Mount St. Mary’s University, Emmitsburg, Maryland, USA; 3 Department of Pathology and Immunology, Washington University School of Medicine, St. Louis, Missouri, USA; 4 Biomolecular NMR Center, The Johns Hopkins University, Baltimore, Maryland, USA

**Keywords:** polyubiquitin, ubiquitin, ubiquitin-binding domain, DNA damage response, alkylation damage, signaling, isothermal titration calorimetry (ITC), nuclear magnetic resonance (NMR), site-directed mutagenesis, immunofluorescence microscopy, ALKBH3, Alpha-ketoglutarate-dependent dioxygenase alkB homolog 3, ASCC2, Activating Signal Cointegrator 1 Complex Subunit 2, CSP, chemical shift perturbation, CUE, coupling of ubiquitin conjugation to ER degradation, HADDOCK, High Ambiguity Driven protein–protein DOCKing, HSQC, Heteronuclear Single Quantum Coherence, ITC, isothermal titration calorimetry, K_d_, equilibrium dissociation constant, MMS, methyl methanesulfonate, TCEP, Tris (2-carboxyethyl) phosphine

## Abstract

Alkylation of DNA and RNA is a potentially toxic lesion that can result in mutations and even cell death. In response to alkylation damage, K63-linked polyubiquitin chains are assembled that localize the Alpha-ketoglutarate-dependent dioxygenase alkB homolog 3–Activating Signal Cointegrator 1 Complex Subunit (ASCC) repair complex to damage sites in the nucleus. The protein ASCC2, a subunit of the ASCC complex, selectively binds K63-linked polyubiquitin chains *via* its coupling of ubiquitin conjugation to ER degradation (CUE) domain. The basis for polyubiquitin-binding specificity was unclear, because CUE domains in other proteins typically bind a single ubiquitin and do not discriminate among different polyubiquitin linkage types. We report here that the ASCC2 CUE domain selectively binds K63-linked diubiquitin by contacting both the distal and proximal ubiquitin. The ASCC2 CUE domain binds the distal ubiquitin in a manner similar to that reported for other CUE domains bound to a single ubiquitin, whereas the contacts with the proximal ubiquitin are unique to ASCC2. Residues in the N-terminal portion of the ASCC2 α1 helix contribute to the binding interaction with the proximal ubiquitin of K63-linked diubiquitin. Mutation of residues within the N-terminal portion of the ASCC2 α1 helix decreases ASCC2 recruitment in response to DNA alkylation, supporting the functional significance of these interactions during the alkylation damage response. Our study reveals the versatility of CUE domains in ubiquitin recognition.

Ubiquitylation is a reversible, posttranslational modification that regulates a vast array of cellular processes including proteasomal degradation, transcription, and the DNA damage response (1, 2, 3). The ubiquitin C-terminus is conjugated to protein substrates in a cascade of enzymatic reactions, most commonly forming a covalent linkage with the ε-amino group of a lysine side chain or the N-terminal α-amine (4). Ubiquitin itself can be ubiquitinated *via* one of its seven lysine residues or at its amino terminus, giving rise to homotypic or branched polyubiquitin chains with distinct topologies and biological functions (5). Different types of polyubiquitin chains are recognized by domains or motifs that bind specifically to the particular ubiquitin modification, thereby recruiting downstream effector proteins (6). In this manner, the diversity of ubiquitin signaling is predicated on the ability of ubiquitin-binding proteins to differentiate among the myriad types of polyubiquitin modifications present in the cell.

Lysine 63 (K63)-linked polyubiquitin chains play a nondegradative role in several DNA damage response pathways, including the response to DNA alkylation (2, 7). The E3 ubiquitin ligase, RNF113A, assembles K63-linked polyubiquitin chains at the sites of alkylation damage (7). These polyubiquitin chains recruit the Alpha-ketoglutarate-dependent dioxygenase alkB homolog 3 (ALKBH3)–ASCC complex, which repairs the lesions (7). A subunit of the complex, ASCC2, binds to the K63-linked polyubiquitin chains *via* its coupling of ubiquitin conjugation to ER degradation (CUE) domain, a ubiquitin-binding domain of approximately 50 amino acids (Fig. 1) (8, 9). As shown for Cue1, Cue2, gp78, and Vps9, CUE domains bind the hydrophobic I44 patch of ubiquitin *via* conserved hydrophobic sequence motifs (Fig. 1*B*) (8, 9, 10, 11). These sequence motifs are conserved in ASCC2, suggesting that its CUE domain binds ubiquitin in a similar manner. Indeed, substitution of ASCC2 residue L506, which lies in the predicted ubiquitin-binding patch, abrogates ubiquitin-binding *in vitro* and dramatically reduces the formation of ASCC2 nuclear foci in response to alkylation damage (7). Coupling of ubiquitin conjugation to ER degradation domains typically make extensive interactions with a single ubiquitin within a polyubiquitin chain and exhibit little selectivity among different types of polyubiquitin chains (8, 9, 10, 12). It is not known what other ASCC2 surfaces mediate interactions with ubiquitin and specify binding for K63-linked polyubiquitin.

**Figure 1:**
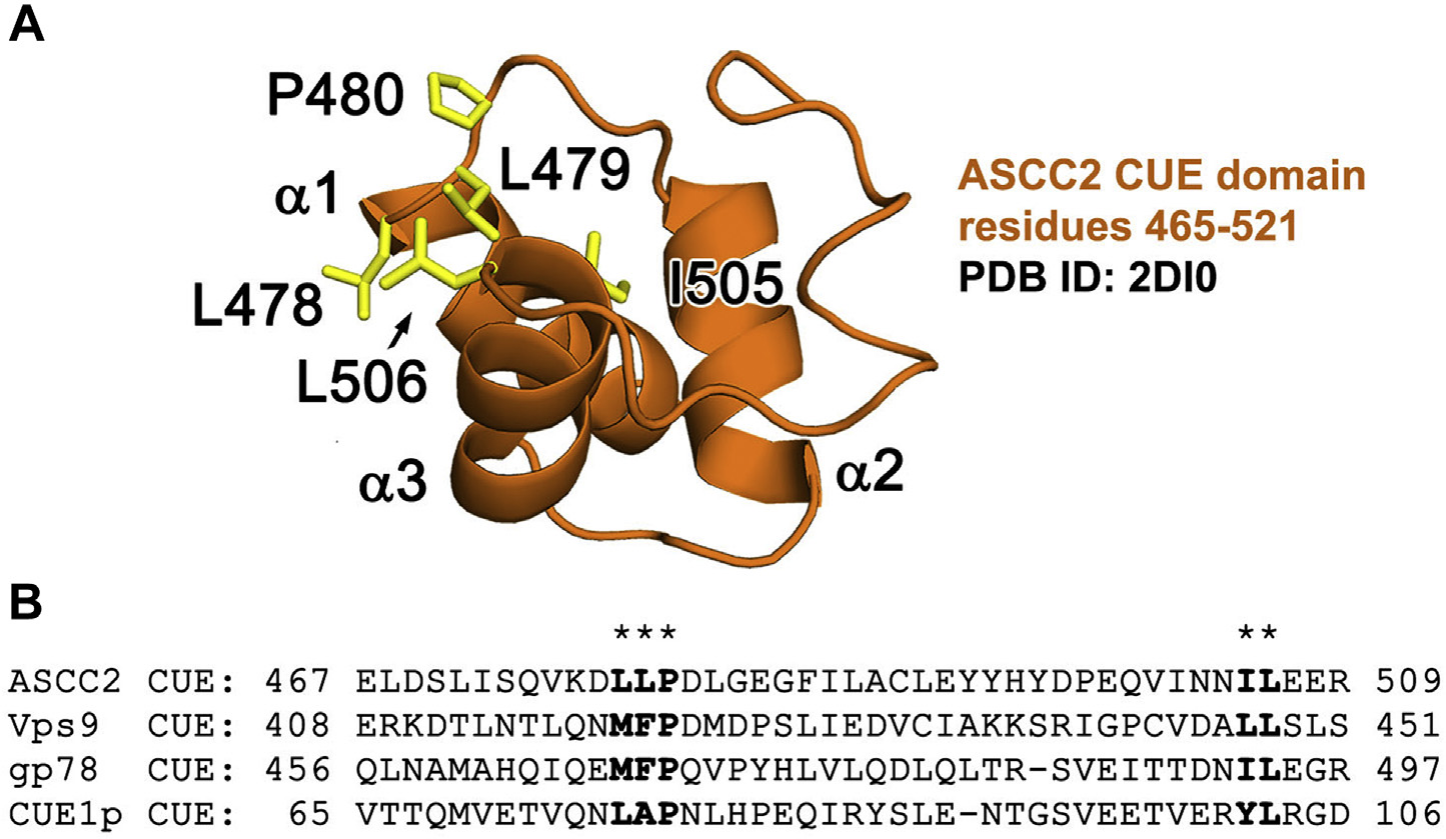
**The ASCC2 CUE domain.***A*, the ASCC2 CUE domain folds into a three-helix bundle. *B*, CUE domains contain conserved sequence motifs on the α1 and α3 helices (in *bold*, below *asterisks*) that form a hydrophobic ubiquitin-binding surface (*yellow sticks* in *A*). ASCC2, Activating Signal Cointegrator 1 Complex Subunit 2; CUE, coupling of ubiquitin conjugation to ER degradation.

We report here that the ASCC2 CUE domain binds with higher affinity to K63-linked polyubiquitin as compared to monoubiquitin or other types of polyubiquitin chains. Using solution NMR, we show that a single ASCC2 CUE domain makes distinct contacts with the two adjacent ubiquitins within a single K63-linked polyubiquitin chain. In addition to mediating conserved interactions with the I44 patch of the distal ubiquitin, a separate region of the ASCC2 CUE domain forms additional interactions with the proximal ubiquitin in K63-linked polyubiquitin. Mutations in the ASCC2 CUE domain residues that contact the proximal ubiquitin disrupt recruitment of ASCC2 to repair foci, consistent with the importance of these residues in binding to K63-linked polyubiquitin. Together, our data show that the ASCC2 CUE domain makes multiple, linkage-specific interactions with adjacent ubiquitins to enhance the affinity of the ALKBH3–ASCC complex for K63-linked polyubiquitin chains at alkylation damage sites.

## Results

### The ASCC2 CUE domain has enhanced affinity and specificity for K63-linked polyubiquitin chains

The ASCC2 CUE domain comprises a three-helix bundle that spans residues 465 to 521 (Fig. 1). The solution structure of the ASCC2 CUE domain has been deposited in the Protein Data Bank (PDB ID: 2DI0) (Fig. 1*A*) and is similar to other experimentally determined CUE domain structures. The structure of the ASCC2 CUE domain superimposes with the Cue2 CUE domain (PDB ID: 1OTR) (8) with an RMSD of 0.92 Å over 37 alpha carbons and with the gp78 CUE domain (PDB ID: 2LVN) (10) with an RMSD of 1.34 Å over 41 alpha carbons. Like other CUE domains, ASCC2 has a cluster of hydrophobic residues on helix 1 and on helix 3, which are predicted to bind to the I44 patch of ubiquitin as in previously characterized CUE:ubiquitin interactions (8, 9, 10, 11, 12) (Fig. 1*B*). The previous finding that a substitution at L506 in helix 3 leads to defects in ASCC2 recruitment in cells (7) is consistent with a role for this surface in ASCC2 binding to ubiquitin.

**Figure 2:**
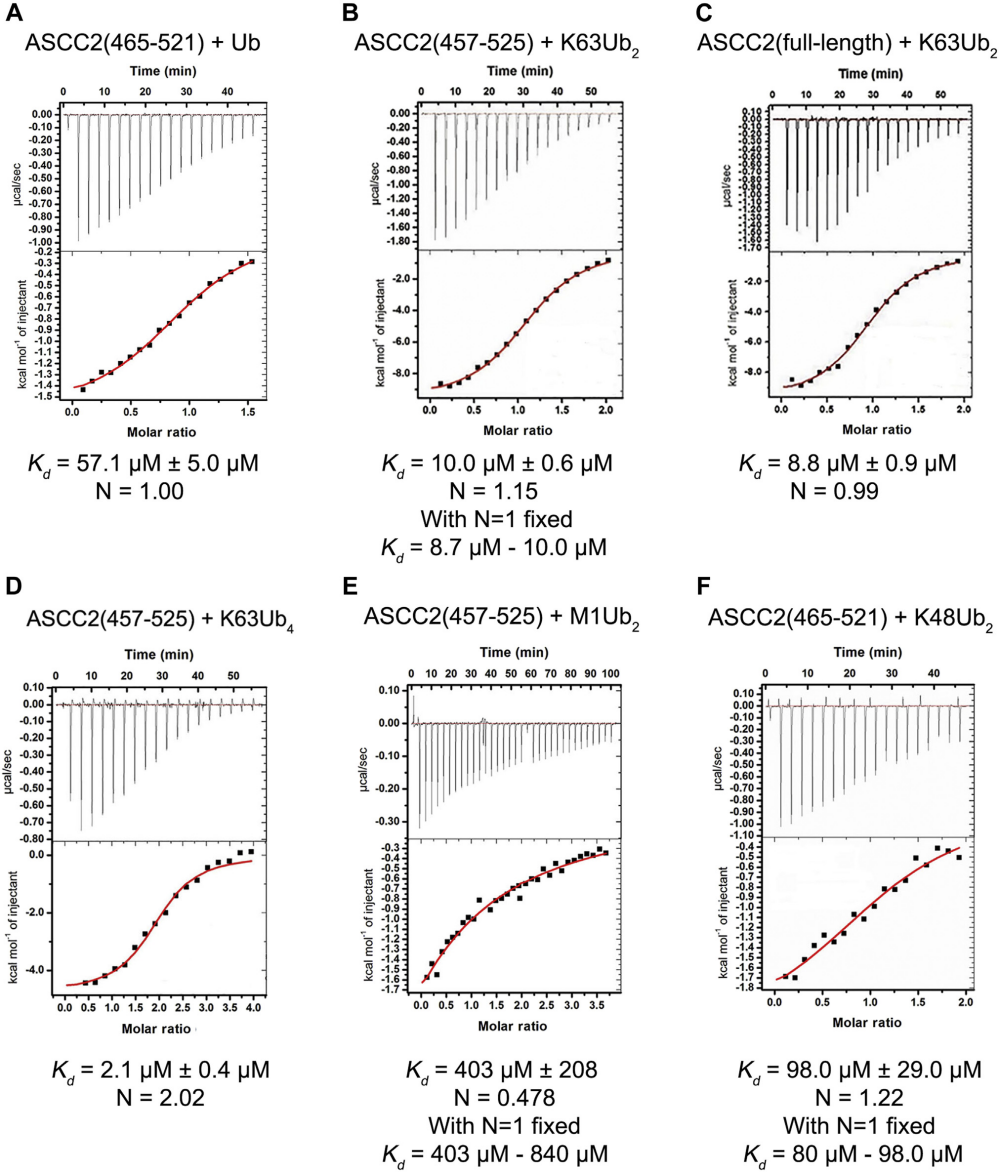
**The ASCC2 CUE domain binds K63Ub**_**2**_**with enhanced affinity and 1:1 stoichiometry.***A*–*C*, ITC data show that full-length ASCC2 and isolated ASCC2 CUE domain bind K63Ub_2_ with greater affinity than monoubiquitin (Ub). *D*, two ASCC2 CUE domains bind per K63-linked tetraubiquitin chain (K63Ub_4_). This 1:1 ASCC2 CUE domain to K63Ub_2_ ratio is observed in *B* and *C* as well. *E*, the ASCC2 CUE domain does not exhibit enhanced-binding affinity for linear diubiquitin (M1Ub_2_) or (*F*) K48-linked diubiquitin (K48Ub_2_). ASCC2, Activating Signal Cointegrator 1 Complex Subunit 2; CUE, coupling of ubiquitin conjugation to ER degradation; ITC, isothermal titration calorimetry.

To determine whether the ASCC2 CUE domain binds in a similar manner to ubiquitin irrespective of whether it is incorporated into a polyubiquitin chain, we used isothermal titration calorimetry (ITC) to compare binding of ASCC2 CUE domain constructs to monoubiquitin and K63-linked diubiquitin (K63Ub_2_). As shown in Figure 2, *A* and *B*, we found that the ASCC2 CUE domain binds with lower affinity to monoubiquitin than to K63Ub_2_. The equilibrium dissociation constant (*K*_d_) for monoubiquitin was 57.1 μM ± 5.0 μM (Fig. 2*A*), whereas ASCC2 bound much more tightly to K63Ub_2_, with a *K*_d_ of 8.7 μM to 10.4 μM (Fig. 2*B*). The affinity of the isolated ASCC2 CUE domain for K63Ub_2_ is similar to that of full-length ASCC2, which binds to K63Ub_2_ with a *K*_d_ of 8.8 μM ± 0.9 μM (Fig. 2*C*). The similar equilibrium dissociation constants suggest that the majority of the affinity comes from the interaction between the CUE domain and K63Ub_2_. Importantly, the approximately 4- to 7-fold enhancement of ASCC2 CUE domain affinity for K63Ub_2_ as compared to monoubiquitin is higher than the 1.0- to 1.8-fold enhancement in affinity that has been reported for other CUE domains binding to polyubiquitin *versus* monoubiquitin (10, 11).

Because very weak binding is difficult to measure accurately by ITC, we also estimated the affinity of the ASCC2 CUE domain for monoubiquitin using NMR spectroscopy. The ^1^H,^15^N-Heteronuclear Single Quantum Coherence (HSQC) spectra of ^15^N-labeled ASCC2(465–521) were recorded in the presence of increasing amounts of monoubiquitin, and the chemical shift perturbations (CSPs) for four ASCC2 residues at the ubiquitin-binding interface were used to calculate the average *K*_d_ value (Fig. S1) (13). The *K*_d_ value of 39.6 μM ± 1.6 μM determined by NMR suggests somewhat tighter monoubiquitin binding than the *K*_d_ determined by ITC (57.1 μM ± 5.0 μM Fig. 2*A*), although still substantially weaker than that measured for K63Ub_2_ (*K*_d_ = 8.7 μM–10.4 μM Fig. 2*B*).

The stoichiometry of the ASCC2 CUE domain binding K63-linked polyubiquitin chains is also different from that of previously studied CUE domains. CUE domains from other ubiquitin-binding proteins, such as gp78, bind diubiquitin with a ratio of two CUE domains per diubiquitin (10), indicating that each CUE domain binds to one ubiquitin in the diubiquitin chain. The ASCC2 CUE domain, however, binds K63Ub_2_ in a 1:1 ratio (Fig. 2*B*), and this ratio is conserved in the binding of full-length ASCC2 to K63Ub_2_ (Fig. 2*C*). Importantly, the observed molar ratio of one ASCC2 CUE domain per K63Ub_2_ is preserved in the context of longer polyubiquitin chains. As shown in Figure 2*D*, the ASCC2 CUE domain binds to K63-linked tetraubiquitin with a molar ratio of 2:1, consistent with each CUE domain binding to two ubiquitins within the tetraubiquitin chain. Interestingly, the affinity of the ASCC2 CUE domain for K63-linked tetraubiquitin is about 4-fold higher than its affinity for diubiquitin (Fig. 2, *B* and *D*).

To test the specificity of ASCC2 for K63-linked diubiquitin as compared to other linkage types, we measured the *K*_d_ of the ASCC2 CUE domain for linear and K48-linked diubiquitin (K48Ub_2_). The affinity of the ASCC2 CUE domain for linear diubiquitin (M1Ub_2_) was extremely weak, with a *K*_*d*_ of about 400 μM (Fig. 2*E*). This result was surprising given that linear and K63-linked polyubiquitin adopt a similar extended topology (14, 15). The affinity of the ASCC2 CUE domain for K48Ub_2_ (Fig. 2*F*), with a *K*_*d*_ of about 98 μM, was similar to that measured for monoubiquitin. These results indicate that the enhanced binding affinity of the ASCC2 CUE domain for polyubiquitin compared to monoubiquitin is specific to K63-linked chains.

### The ASCC2 CUE domain forms different contacts with the distal and proximal ubiquitin of K63Ub_2_

**Figure 3:**
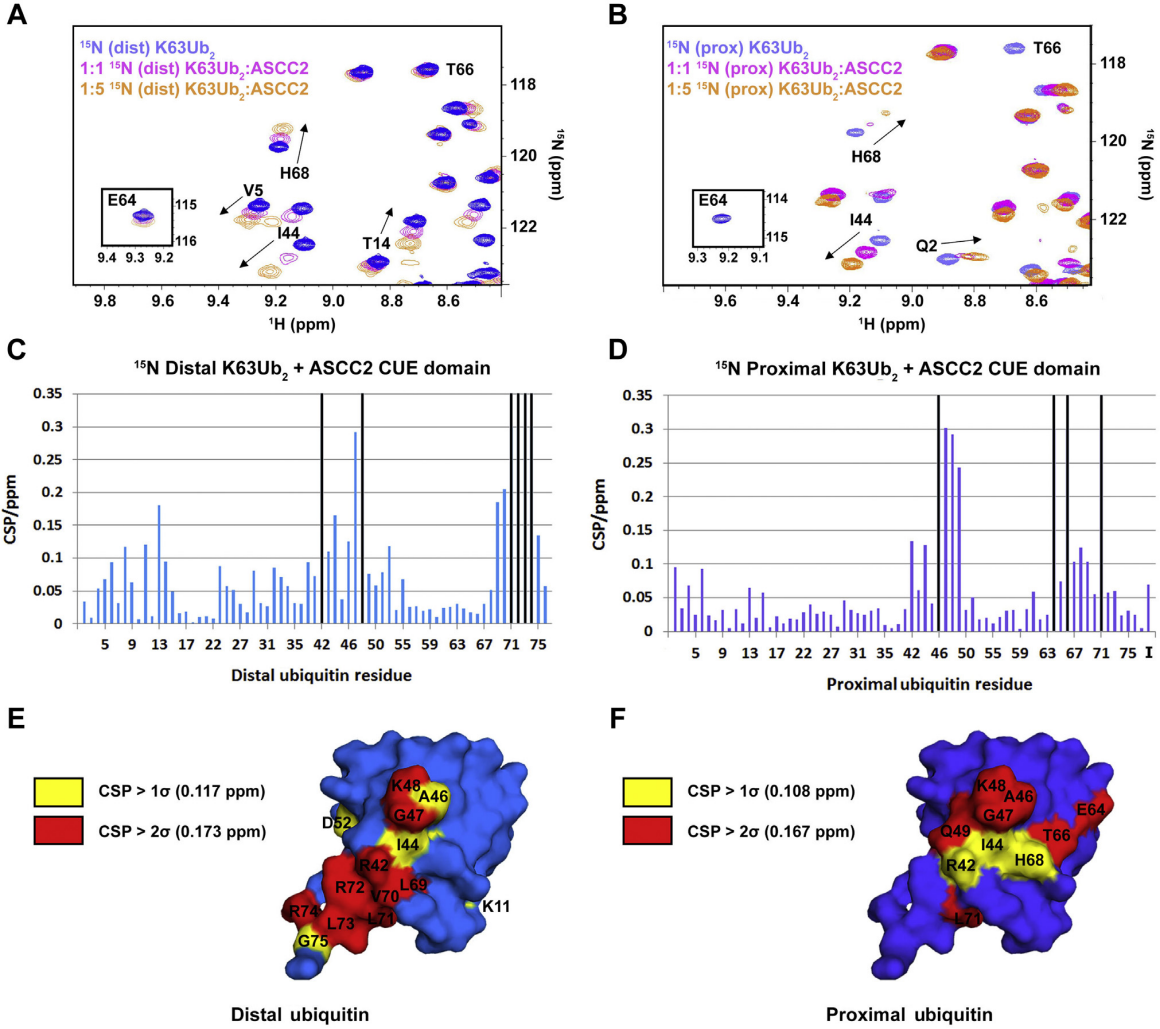
**The ASCC2 CUE domain makes different interactions with the distal and proximal ubiquitins of K63Ub**_**2**_**.** Regions of the ^1^H,^15^N-HSQC spectra for the ^15^N-labeled distal (*A*) and proximal (*B*) ubiquitins of K63Ub_2_ titrated with the ASCC2 CUE domain suggest different residues from each ubiquitin contact ASCC2. For example, the resonances for residues E64 and T66 disappear during the titration with the ASCC2 CUE domain for the ^15^N-labeled proximal ubiquitin (*B*) but show modest shifts for the ^15^N-labeled distal ubiquitin (*A*). The CSPs values for all residues from ^15^N-labeled distal and proximal ubiquitins of K63Ub_2_ titrated with the ASCC2 CUE domain are reported in panels *C* and *D*, respectively. *Black bars* indicate the resonance disappeared during the titration, and the “I” in *D* denotes the K63Ub_2_ isopeptide bond. Highlighting residues with CSP values greater than 1σ (*yellow*) and 2σ (*red*) on the surface of the distal (*E*) and proximal (*F*) ubiquitins suggest that ASCC2 makes different contacts with the distal and proximal ubiquitins of K63Ub_2_. ASCC2, Activating Signal Cointegrator 1 Complex Subunit 2; CSP, chemical shift perturbation; CUE, coupling of ubiquitin conjugation to ER degradation; HSQC, Heteronuclear Single Quantum Coherence.

The higher affinity of ASCC2 for diubiquitin or tetraubiquitin and the molar ratio of one ASCC2 CUE domain per diubiquitin are consistent with a single CUE domain simultaneously contacting the linked proximal and distal ubiquitin. Given the small size and asymmetric fold of the CUE domain, ASCC2 would need to form different binding interfaces with the two ubiquitin monomers. We used NMR chemical shift mapping experiments to compare ASCC2 CUE domain contacts with the distal and proximal ubiquitins of K63Ub_2_. To distinguish the two covalently linked ubiquitin monomers, we generated diubiquitin with either the proximal or the distal ubiquitin isotopically labeled with ^15^N. The ^1^H,^15^N-HSQC spectra of the differently labeled K63Ub_2_ were recorded in the presence of increasing concentrations of the ASCC2 CUE domain (Fig. 3, *A* and *B*). The CSP values for the ^15^N-labeled distal ubiquitin of K63Ub_2_ titrated with the ASCC2 CUE domain (Fig. 3*C*) are similar to those reported for other CUE domains interacting with monoubiquitin (10, 11). The common features include relatively large CSP values for residues in and around the I44 patch, such as R42, I44, G47, and K48, and for residues 70 to 74 at the C-terminal tail of ubiquitin. The similar CSP values suggest the distal ubiquitin of K63Ub_2_ binds the ASCC2 CUE domain using the same surface as previously reported CUE:ubiquitin interactions (10, 11).

The CSP values for the ^15^N-labeled proximal ubiquitin of K63Ub_2_ titrated with ASCC2(465–521) (Fig. 3*D*), however, were markedly different from those observed for the ^15^N-labeled distal ubiquitin. The CSP values for ubiquitin residues V70, R72, L73, and R74 were smaller in the experiments with ^15^N-labeled proximal ubiquitin (Fig. 3*D*) as compared to the experiments with ^15^N-labeled distal ubiquitin (Fig. 3*C*). In addition, large CSP values for residues E64 and T66 (Fig. 3*D*) were unique to the proximal ubiquitin. The large CSP values for proximal ubiquitin residues E64 and T66 and the small CSP values for residues in the ubiquitin C-terminal tail suggest that the ASCC2 CUE domain contacts the proximal ubiquitin in a noncanonical manner. The differences between the distal and proximal ubiquitin CSP values suggest that the two ubiquitins use different surface residues to interact with the ASCC2 CUE domain (Fig. 3, *E* and *F*).

**Figure 4:**
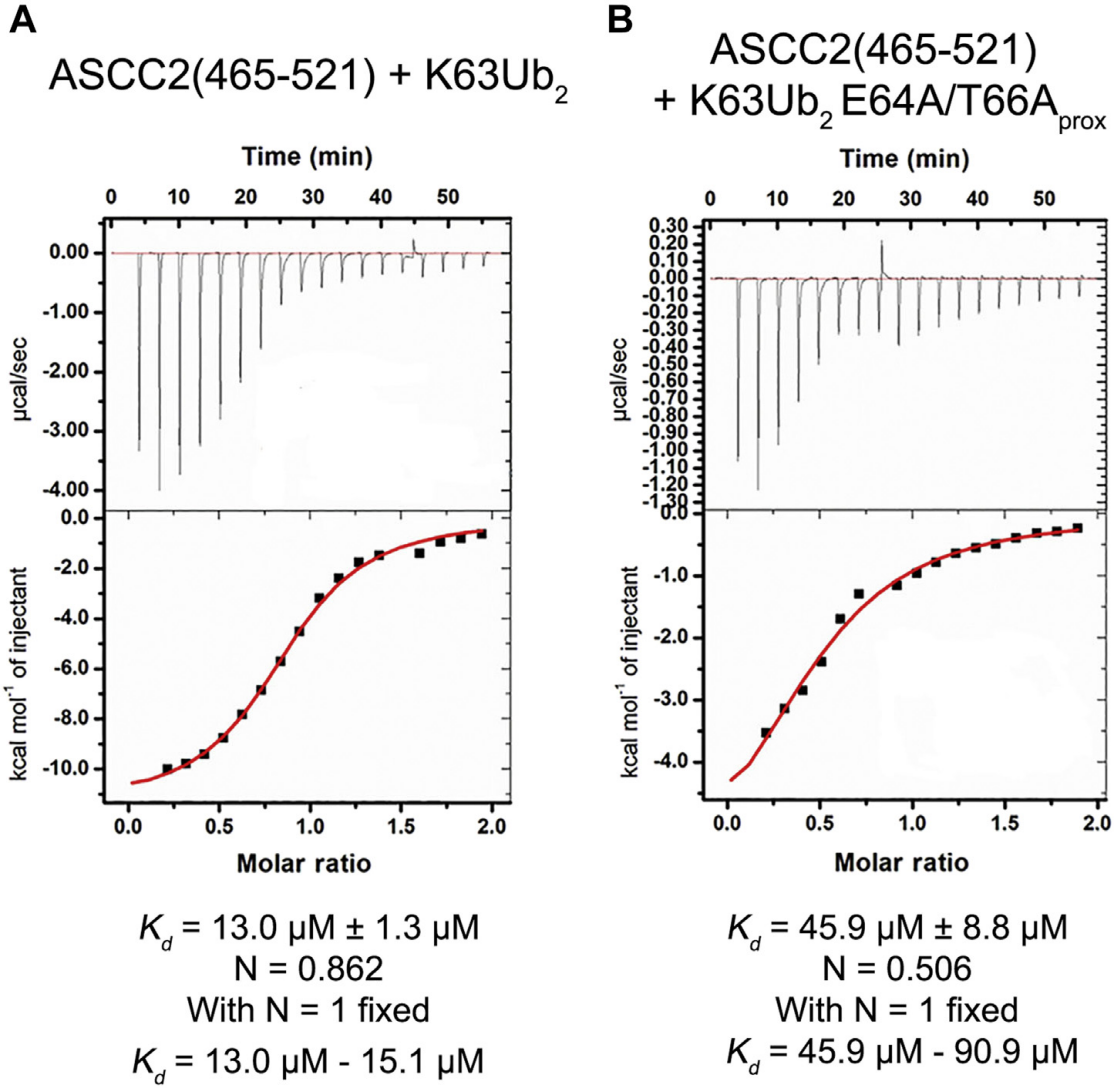
**The ASCC2 CUE domain binds K63Ub**_**2**_**E64A/T66A**_**prox**_**with reduced affinity relative to WT K63Ub**_**2**_**.** ITC data show that ASCC2(465–521) binds WT K63Ub_2_ (*A*) with ∼3.5 to 7.0× greater affinity than K63Ub_2_ E64A/T66A_prox_ (*B*). ASCC2, Activating Signal Cointegrator 1 Complex Subunit 2; CUE, coupling of ubiquitin conjugation to ER degradation; ITC, isothermal titration calorimetry.

To determine the contribution of proximal ubiquitin residues E64 and T66 to ASCC2 CUE domain binding, we measured the affinity of the ASCC2 CUE domain for K63Ub_2_ bearing side chains substitutions at proximal ubiquitin residues E64 and T66 (K63Ub_2_ E64A/T66A_prox_) using ITC. As shown in Figure 4, the ASCC2 CUE domain binds K63Ub_2_ E64A/T66A_prox_ with a *K*_d_ in the range of 45.9 μM to 90.9 μM, approximately 3.5 to 7.0 times more weakly than WT K63Ub_2_. This result supports the NMR data (Fig. 3*B*) in suggesting that the ASCC2 CUE domain interacts with residues E64 and T66 of the proximal ubiquitin in K63Ub_2_.

### The N-terminal portion of the ASCC2 α1 helix is important for K63-linked polyubiquitin binding and recruitment to DNA damage foci

**Figure 5:**
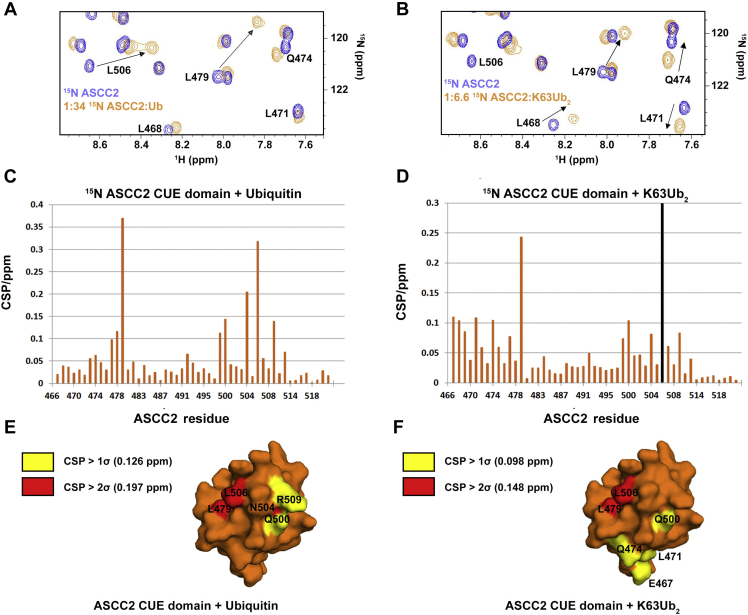
**The ASCC2 CUE domain uses conserved sequences from the α1 and α3 helices, along with the N-terminal end of the α1 helix, to bind K63-linked polyubiquitin chains.** Regions of the ^1^H-^15^N-HSQC spectra of ^15^N-labeled ASCC2(465–521) show that residues from the ASCC2 α1 helix (*e.g.*, L468, L471, and Q474) exhibit larger CSPs when titrated with K63Ub_2_ (*B*) compared to monoubiquitin (*A*). CSP values recorded for all ^15^N-labeled ASCC2(465–521) residues titrated with monoubiquitin or K63Ub_2_ are reported in Figure 5, *C* and *D*, respectively. A *black bar* indicates the resonance disappeared during the titration. Coloring ASCC2(465–521) surface residues with CSP values greater than 1σ (*yellow*) and 2σ (*red*) in the presence of monoubiquitin (*E*) and K63Ub_2_ (*F*) suggests that the N-terminal portion of the ASCC2 α1 helix may form a second ubiquitin-binding site for interacting with K63-linked polyubiquitin chains. ASCC2, Activating Signal Cointegrator 1 Complex Subunit 2; CSP, chemical shift perturbation; CUE, coupling of ubiquitin conjugation to ER degradation; HSQC, Heteronuclear Single Quantum Coherence.

To determine which ASCC2 residues interact with K63-linked polyubiquitin, the ^1^H,^15^N-HSQC spectra of ^15^N-labeled ASCC2 CUE domain were recorded in the presence of increasing concentrations of monoubiquitin and K63Ub_2_ (Fig. 5). ASCC2 residues L479 and L506, from the conserved CUE domain hydrophobic sequence motifs in the α1 and α3 helices, respectively, exhibited large CSP values when the ASCC2 CUE domain was titrated with monoubiquitin or K63Ub_2_ (Fig. 5, *A*–*D*). This finding suggests that, as with other CUE domains (8, 9, 10, 11, 12), the hydrophobic surface created by the conserved sequence motifs is the binding site for monoubiquitin and one of the ubiquitins in K63Ub_2_. The distal ubiquitin of K63Ub_2_ is most likely to bind the hydrophobic surface formed by the conserved sequence motifs given the similarities between its CSP values (Fig. 3*C*) and those reported for monoubiquitin titrated with other CUE domains (10, 11).

The ASCC2 residues that interact with the proximal ubiquitin of K63-linked diubiquitin would be expected to have larger CSP values when the CUE domain is titrated with K63Ub_2_ than when it is titrated with monoubiquitin. The α1 helix (residues 465–479; Fig. 1*A*) is the only region of the ASCC2 CUE domain that has dramatically different CSP values in the presence of K63Ub_2_ compared to monoubiquitin (Fig. 5, *A*–*D*). The majority of residues from the N-terminal end of the α1 helix have relatively small CSP values when titrated with monoubiquitin (Fig. 5, *A* and *C*). When titrated with K63Ub_2_, however, the CSP values for residues from the N-terminal end of the α1 helix are larger (Fig. 5, *B* and *D*). The increased CSP values for residues at the N-terminal end of the α1 helix in the presence of K63Ub_2_ compared to monoubiquitin suggest that these residues may form a second binding site for the proximal ubiquitin of K63Ub_2_ (Fig. 5, *E* and *F*).

**Figure 6:**
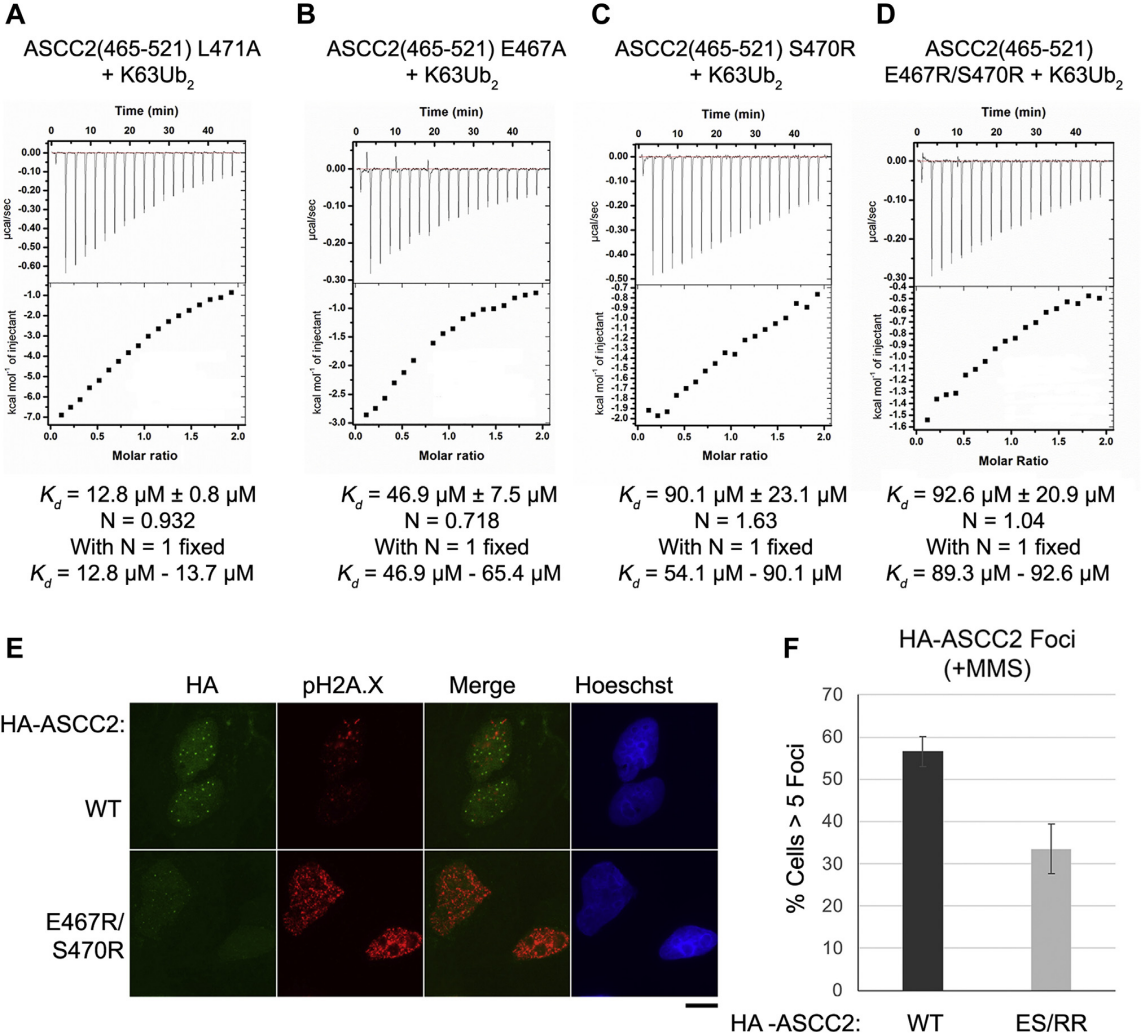
**ASCC2 residues E467 and S470 bind the proximal ubiquitin of K63Ub**_**2**_**.***A*, the ASCC2(465–521) L471A mutant binds K63Ub_2_ with nearly the same affinity as WT ASCC2(465–521) (Fig. 4*A*). *B*, the ASCC2(465–521) E467A mutant, however, binds K63Ub_2_ approximately 3.6- to 5.0-fold more weakly than WT ASCC2(465–521) and (*C*) the ASCC2(465–521) S470R mutant, and (*D*) the ASCC2(465–521) E467R/S470R double mutant bind approximately 7.0-fold more weakly. These results suggest that ASCC2 residues E467 and S470 participate in the binding interaction with K63-linked polyubiquitin chains. *E*, HA-tagged ASCC2, or the E467R/S470R mutant, were expressed in U2OS cells, then treated with 0.5 mM MMS for 6 h. Immunofluorescence for HA and pH2A.X were performed after extraction with Triton X-100, as shown, with Hoechst used as the nuclear counter stain. The scale bar represents 10 μm. *F*, quantification of foci formation. N = 3 replicates and the error bars indicate ±S.D. of the mean. ASCC2, Activating Signal Cointegrator 1 Complex Subunit 2; MMS, methyl methanesulfonate.

To test the hypothesis that ASCC2 residues at the N-terminal end of the α1 helix bind the proximal ubiquitin of K63Ub_2_, we made point mutations at ASCC2 residues E467, S470, and L471 and assayed their effects on ASCC2 CUE domain binding to K63Ub_2_. Although ITC experiments showed that the affinity of the ASCC2(465–521) L471A mutant for K63Ub_2_ was nearly identical to that of WT ASCC2(465–521) (Fig. 6*A*), the ASCC2 E467A mutant bound 3.6- to 5.0-fold more weakly, with a *K*_d_ in the range of 46.9 μM to 65.4 μM (Fig. 6*B*). The ASCC2 S470R mutant bound with even lower affinity, with an apparent *K*_d_ of 90.9 μM ± 23.1 μM (Fig. 6*C*). An ASCC2 E467R/S470R double mutant bound K63Ub_2_ with an apparent *K*_d_ of 92.6 μM ± 20.9 μM (Fig. 6*D*). The decrease in K63Ub_2_ binding affinity observed upon mutating the α1 helix stands in contrast to the effect observed upon altering other ASCC2 CUE domain regions that could potentially interact with the proximal ubiquitin of K63Ub_2_, such as the α2 helix, or the loop connecting the α2 and α3 helices, where mutations resulted in little change in binding affinity (Fig. S2). The decreased affinity observed for the E467 and S470 mutant proteins is consistent with ^1^H,^15^N-HSQC data (Fig. 5) suggesting that the ASCC2 CUE domain binds the proximal ubiquitin of K63Ub_2_ using a second, previously uncharacterized, interaction site located at the N-terminal end of the α1 helix. The presence of a second binding site on the ASCC2 CUE domain could account for the enhanced affinity of the ASCC2 CUE domain for K63Ub_2_ relative to monoubiquitin and the 1:1 stoichiometry of ASCC2 CUE:K63Ub_2_ binding (Fig. 2).

To test the functional importance of the N-terminal portion of the ASCC2 CUE domain α1 helix in cells, we studied the effect of an E467R/S470R double mutation on the recruitment of ASCC2 to alkylation damage-induced foci. As compared to WT ASCC2, the E467R/S470R double mutant had significantly reduced ASCC2 foci after alkylation damage was induced with methyl methanesulfonate (MMS) (Fig. 6, *E* and *F*). This mutant was expressed at levels similar to the WT protein, suggesting that the defect is not because of a loss of expression due to misfolding or other global defect (Fig. S3). These results are consistent with a role for the N-terminal portion of the ASCC2 CUE domain α1 helix in its recruitment during the DNA damage response.

### Model of the ASCC2 CUE domain binding to K63-linked diubiquitin

**Figure 7:**
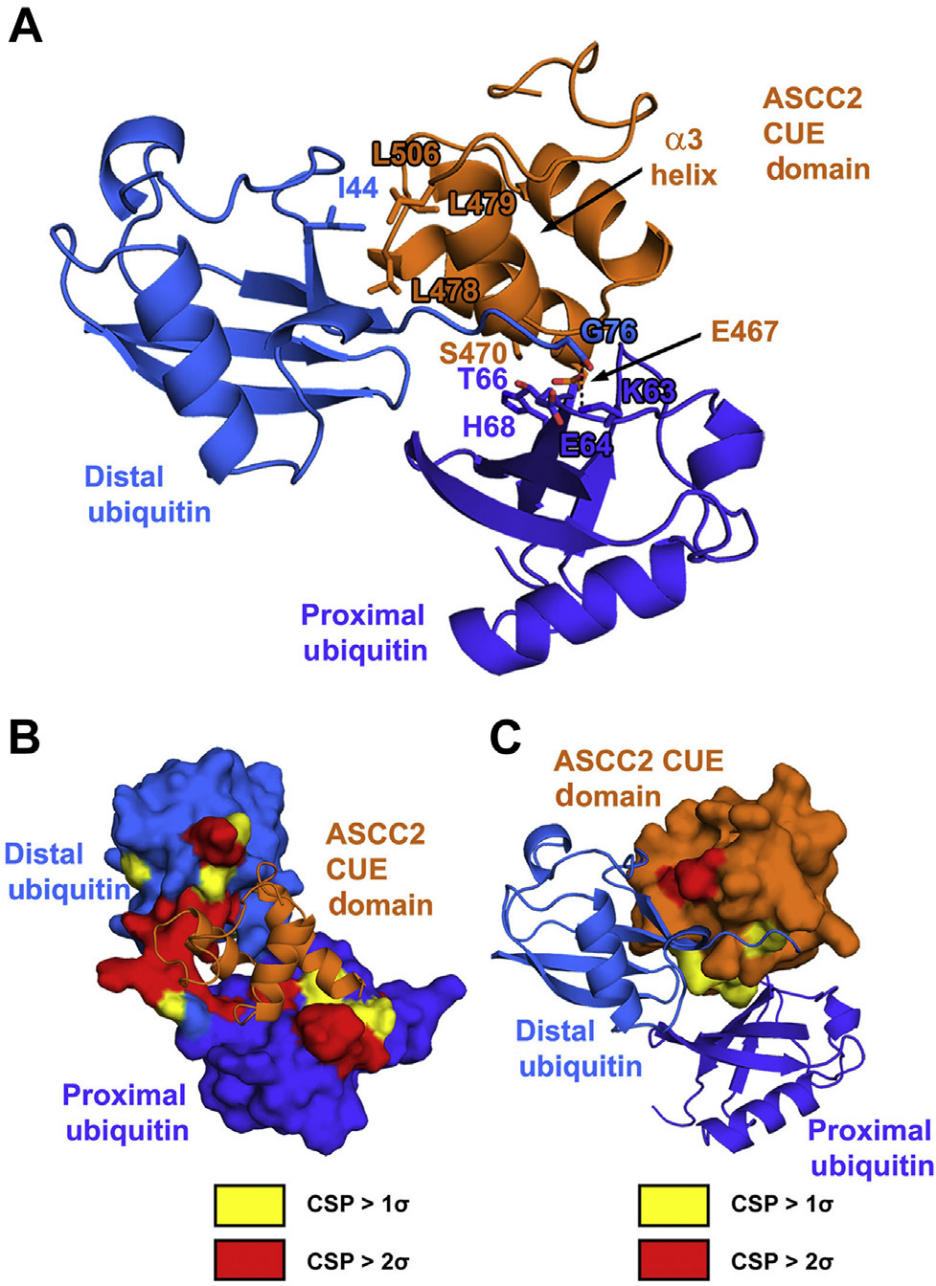
**Model of the ASCC2(465–521):K63Ub**_**2**_**complex.***A*, the ASCC2 CUE domain was superimposed onto the gp78 CUE domain structure in the gp78 CUE:ubiquitin complex (PDB ID: 2LVO). The proximal ubiquitin of K63Ub_2_ was docked to the complex using the HADDOCK server. In this model, binding of the I44 patch of the distal ubiquitin to the hydrophobic α1 and α3 sequence motifs of ASCC2, mediated by residues including L479 and L506, positions the C-terminal tail of the distal ubiquitin to interact with the α3 helix of ASCC2. Isopeptide bond formation at proximal ubiquitin residue K63 (*dashed line*) places adjacent proximal ubiquitin residues E64 and T66 within binding distance of the ASCC2 CUE domain. The proximal ubiquitin of K63Ub_2_ docks against the N-terminal end of the CUE domain α1 helix, where ASCC2 residues E467 and S470 interact with proximal ubiquitin residues. In *B* and *C*, the model of the ASCC2(465–521):K63Ub_2_ complex is shown with K63Ub_2_ or ASCC2(465–521) in surface representation, respectively. The residues with CSP values greater than 1σ are colored *yellow* and residues with CSP values greater than 2σ are colored *red*, according to the data in Figures 3, *C* and *D* and 5*D*. ASCC2, Activating Signal Cointegrator 1 Complex Subunit 2; CSP, chemical shift perturbation; CUE, coupling of ubiquitin conjugation to ER degradation; HADDOCK, High Ambiguity Driven protein–protein DOCKing.

We modeled the interaction between the proximal ubiquitin of K63Ub_2_ and the ASCC2 CUE domain using the High Ambiguity Driven protein–protein DOCKing (HADDOCK) protein-docking server (16, 17). We first generated a model of the interaction between the ASCC2 CUE domain and the distal ubiquitin based on the gp78 CUE:monoubiquitin complex (PDB ID: 2LVO) (10) by superimposing residues 465 to 521 of the ASCC2 CUE domain structure (PDB ID: 2DI0) on the gp78 CUE domain. Given the similarity between the CSP values for the distal ubiquitin of K63Ub_2_ titrated with the ASCC2 CUE domain (Fig. 3*C*) and the CSP values for monoubiquitin titrated with the gp78 CUE domain (10), it is likely that these interactions are structurally similar. Distance restraints based on NMR CSP data and mutagenesis data were used by the HADDOCK server to guide the docking of the proximal ubiquitin of K63Ub_2_ to the ASCC2 CUE domain. ASCC2 residues E467 and S470, and proximal ubiquitin residues E64 and T66, were specified as residues likely to be at the binding interface based on the deleterious effect of substitutions at these residues on binding (Figs. 4*B* and 6, *B*–*D*). In addition, proximal ubiquitin residues with CSP values greater than 2σ were also specified as likely to be at the interface with ASCC2. These proximal ubiquitin residues include A46, G47, K48, Q49, and L71. The resulting model of the ASCC2(465–521):K63Ub_2_ complex is shown in Figure 7*A*. This model places the majority of the K63Ub_2_ and ASCC2(465–521) residues with the largest CSP values at the binding interface, as shown in Figure 7, *B* and *C*, respectively.

## Discussion

The preferential binding of ASCC2 to K63-linked polyubiquitin chains stands in contrast to other CUE domain proteins, which show more modest enhancement of binding to monoubiquitin *versus* polyubiquitin and little selectivity among polyubiquitin chain types (10, 12). We found that the affinity of the ASCC2 CUE domain for K63Ub_2_ is approximately 4- to 7-fold higher than its affinity for monoubiquitin, and that the ASCC2 CUE domain binds to diubiquitin and tetraubiquitin in a ratio of one CUE domain per diubiquitin (Fig. 2). Interactions with the distal ubiquitin are similar to those observed for other CUE domains bound to monoubiquitin (8, 9, 10, 11, 12), whereas ASCC2 contacts the proximal ubiquitin in a noncanonical manner using residues from the N-terminal portion of the α1 helix (Figs. 5 and 6). By contrast, CUE domains from other proteins, such as Cue1 and gp78, make fewer contacts with adjacent ubiquitins within polyubiquitin chains and, accordingly, exhibit smaller enhancements in their affinities for polyubiquitin chains compared to monoubiquitin (10, 11). For example, the CUE domain from the protein Cue1 binds to the I44 patch of the proximal ubiquitin in K48Ub_2_ while also contacting G75 and the C-terminus of the distal ubiquitin (11). The *K*_d_ for the Cue1 CUE domain binding K48Ub_2_ is 95 μM compared to 173 μM for binding to monoubiquitin (11). This enhancement is much more modest than the 4- to 7-fold enhancement observed for the ASCC2 CUE domain. The gp78 CUE domain contacts T66 of the proximal ubiquitin in K48Ub_2_ while binding the I44 patch of the distal ubiquitin, resulting in an enhancement in affinity for the distal ubiquitin in K48Ub_2_ relative to the proximal ubiquitin (10). Overall, however, the gp78 CUE domain binds K48Ub_2_ and monoubiquitin with virtually equal affinity of about 12.4 μM and 12.8 μM, respectively (10). Despite the modest enhancement in affinity for polyubiquitin chains exhibited by the gp78 and Cue1 CUE domains, interacting with adjacent ubiquitins simultaneously is important for their biological functions. For these CUE domains, the interactions described above properly position ubiquitin ligases to add to growing polyubiquitin chains (10, 11). For the ASCC2 CUE domain, interactions with adjacent ubiquitins strengthen the affinity for ASCC2’s biologically relevant target, K63-linked polyubiquitin chains (Fig. 2), and increase the recruitment of the ALKBH3–ASCC repair complex to alkylation damage sites (Fig. 6, *E* and *F*).

Although the ASCC2 CUE domain has been shown to bind K63Ub_2_ with enhanced affinity relative to M1Ub_2_ and K48Ub_2_, the structural basis for this selectivity has not been fully elucidated. Linear polyubiquitin chains, and possibly K48-linked polyubiquitin chains, can adopt similar conformations to the K63-linked polyubiquitin chain shown in Figure 7 (14, 15) and interact with the same surfaces of the ASCC2 CUE domain. However, the affinity of the ASCC2 CUE domain for M1Ub_2_ and K48Ub_2_ relative to K63Ub_2_ is much weaker (Fig. 2). We speculate that ASCC2 CUE domain interactions with proximal ubiquitin residues near the K63 isopeptide linkage, including residues E64 and T66, contribute to the selectivity for K63-linked polyubiquitin. Consistent with this, we found that alanine substitutions of proximal ubiquitin residues E64 and T66 reduced the affinity for K63Ub_2_ by about 3.5- to 7.0-fold (Fig. 4). Other ubiquitin-binding domains specific for K63-linked polyubiquitin, such as the myosin VI MyUb domain, similarly contact the I44 patch of the distal ubiquitin and proximal ubiquitin residues near the K63 isopeptide linkage, including T66 (18), suggesting that this may be a general mechanism for imparting specificity. Furthermore, it is not known which proximal ubiquitin surface in K63Ub_2_ interacts with the N-terminal portion of the ASCC2 CUE domain α1 helix and whether the proximal ubiquitins of M1Ub_2_ and K48Ub_2_ are capable of making similar contacts. Elucidating the structural details of the interactions between K63Ub_2_ residues E64, T66, and the proximal ubiquitin as a whole, with the ASCC2 CUE domain will be the subject of continued investigation of the basis for ASCC2 domain selectivity.

## Experimental procedures

### Plasmids for protein expression

ASCC2 constructs containing amino acids 457 to 525 or 465 to 521 were inserted into a pPROEX HTa vector with an N-terminal polyhistidine tag followed by a tobacco etch virus protease recognition sequence. Full-length ASCC2 was inserted into the pET28 vector. WT ubiquitin residues 1 to 76 (wt Ub), along with mutant ubiquitin constructs containing K48R/K63R substitutions (K48R/K63R Ub) or a D77 extension (D77 Ub), were inserted into the pET-3a vector.

### Plasmids for cell-based studies

Full-length human ASCC2 cDNA cloned into pENTR-3C and pET-28a-Flag was previously described (7). The ASCC2 E467R/S470R mutant cDNA was synthesized (IDT), cloned into pENTR-3C and pET-28a-Flag, and confirmed by Sanger sequencing. For human cell expression, ASCC2 E467R/S470R was subcloned into pHAGE-CMV-3XHA using Gateway recombination.

### Expression and purification of the ASCC2 CUE domain

BL21 DE3 *Escherichia coli* cells were transformed with pPROEX HTa vector containing the ASCC2 constructs, plated on LB agar containing 100 μg/ml ampicillin, and incubated overnight at 37 °C. Single colonies were used to inoculate 5-ml aliquots of LB media with 100 μg/ml ampicillin. The 5-ml cultures were grown overnight at 37 °C with 250 rpm shaking until saturation. The 5-ml colonies were used to inoculate 1-l cultures of LB media with 100 μg/ml ampicillin, which were grown at 37 °C with 250 rpm shaking until reaching an A_600_ between 0.5 and 0.8. Protein expression was induced by adding 0.5 mM IPTG and allowed to continue overnight at 16 °C with 250 rpm shaking. After protein expression, the cells were pelleted by centrifugation, resuspended in a buffer consisting of 50 mM Tris pH 7.5 and 1 mM PMSF, and lysed by sonication on ice. Cell lysate was centrifuged at 17,500*g* for 30 min at 4 °C, passed through a filter with 0.22 μm pore size, and loaded onto a 5-ml HisTrap column (Cytiva life sciences) that had been equilibrated in buffer A (50 mM Tris pH 7.5, 250 mM NaCl, 10 mM imidazole, and 200 μM Tris (2-carboxyethyl) phosphine (TCEP)). The His-tagged ASCC2 CUE domain that was retained by the HisTrap column was eluted by running a gradient from 0% to 100% buffer B (50 mM Tris pH 7.5, 250 mM NaCl, 400 mM imidazole, and 200 μM TCEP) over 100 ml. The fractions judged to contain His-tagged ASCC2 CUE domain by gel electrophoresis were combined and incubated with His-tagged tobacco etch virus protease while being dialyzed in buffer A overnight at 4 °C. The dialyzed sample was then repassed over a HisTrap column equilibrated in buffer A, and the flowthrough containing the untagged ASCC2 CUE domain was collected and concentrated to less than 5 ml. The concentrated ASCC2 CUE domain solution was passed over a Superdex 75 16/60 size-exclusion column (Cytiva life sciences) equilibrated in 20 mM Hepes pH 7.6, 150 mM NaCl, and 200 μM TCEP. The ASCC2 CUE domain eluted from the column as a single peak roughly 85 ml after injection.

### Expression and purification of full-length ASCC2

*E. coli* Rosetta (DE3) cells were transformed with pET-28 vector containing full-length ASCC2 and grown on LB agar plates with kanamycin and chloramphenicol. The resulting colonies were used to inoculate 5 ml cultures of LB media with kanamycin and chloramphenicol, which were grown overnight at 37 °C and 250 rpm shaking until reaching saturation. The 5-ml cultures were used to inoculate 1-l cultures of LB media with kanamycin and chloramphenicol that were grown at 37 °C and 250 rpm shaking until reaching an A_600_ between 0.5 and 0.8. Once the cells had reached the appropriate density, the temperature was lowered to 16 °C, and ASCC2 expression was induced by adding 500 μl of 1 M IPTG. After approximately 16 h, the cells were harvested by centrifuging at 5000 rpm for 20 min. The cells were resuspended in 100 ml of lysis buffer (50 mM Tris pH 7.5, 250 mM NaCl, 20 mM imidazole, 3 mM β-mercaptoethanol, 2 μM PMSF, and one cOmplete Mini, EDTA-free protease-inhibitor tablet (Roche)), lysed using a microfluidizer, and then centrifuged at 14,000 rpm for 30 min at 4 °C to separate the soluble and insoluble fractions. The soluble fraction was then passed through syringe filters with 0.45-micron and 0.22-micron pore sizes before being loaded onto a 5-ml HisTrap column (Cytiva life sciences) that had been equilibrated in buffer A (50 mM Tris pH 7.5, 250 mM NaCl, 20 mM imidazole, and 3 mM β-mercaptoethanol). His-tagged, full-length ASCC2 was eluted from the column using a gradient from 0% to 100% buffer B (50 mM Tris pH 7.5, 250 mM NaCl, 400 mM imidazole, and 3 mM β-mercaptoethanol) over 50 ml. Fractions containing full-length ASCC2, as determined by SDS-PAGE, were concentrated to less than 5 ml total volume and passed over a Superdex 200 16/60 size-exclusion column (Cytiva Life Sciences) that had been equilibrated in a buffer consisting of 20 mM Hepes pH 7.5, 150 mM NaCl, and 200 μM TCEP. Full-length ASCC2 eluted from the Superdex 200 16/60 size-exclusion column 60 to 70 ml after injection.

### Expression and purification of monoubiquitin

BL21 DE3 *E. coli* cells were transformed with pET-3a vector containing the ubiquitin constructs, plated on LB agar containing 100 μg/ml ampicillin, and incubated overnight at 37 °C. Single colonies were used to inoculate 5-ml aliquots of LB media with 100 μg/ml ampicillin. The 5-ml cultures were grown overnight at 37 °C with 250 rpm shaking until saturation. The 5-ml colonies were used to inoculate 1-l cultures of LB media with 100 μg/ml ampicillin, which were grown at 37 °C with 250 rpm shaking until reaching an A_600_ between 0.5 and 0.8. Protein expression was induced by adding 0.5 mM IPTG and allowed to continue overnight at 16 °C with 250 rpm shaking. After protein expression, the cells were pelleted by centrifugation, resuspended in a buffer consisting of 50 mM Tris pH 7.5 and 1 mM PMSF, and lysed by sonication on ice. Cell lysate was centrifuged at 17,500*g* for 30 min at 4 °C, after which the soluble fraction was separated and slowly stirred on ice. To the stirring soluble fraction, 1% (v/v) of 70% perchloric acid was added dropwise until the solution turned a milky white. This solution was centrifuged at 17,500*g* for 30 min at 4 °C, after which the soluble fraction containing the ubiquitin was separated from the pellet. The soluble fraction was then subjected to multiple rounds of dialysis in 10 mM Tris pH 7.6 until reaching a neutral pH.

### Conjugation and purification of polyubiquitin chains

Polyubiquitin chains were assembled enzymatically by combining monoubiquitin (>1 mM), human UBE1 enzyme (500 nM), and *Saccharomyces cerevisiae* Ubc13/Mms2 (2.5 μM) in a solution containing 50 mM Hepes pH 7.5, 10 mM MgCl_2_, 1 mM TCEP, and 10 mM ATP. To limit the chain length to diubiquitin, K48R/K63R ubiquitin and D77 ubiquitin can be substituted for WT ubiquitin in the reaction mixture (19). K48R/K63R ubiquitin will occupy the distal position in K63Ub_2_, and D77 ubiquitin will occupy proximal position in K63Ub_2_. Human UBE1 and *S. cerevisiae* Ubc13/Mms2 enzymes were expressed and purified, as previously described (20, 21). The reaction mixture was incubated overnight at 37 °C and then diluted 10-fold in buffer A (50 mM ammonium acetate pH 4.5 and 50 mM NaCl) and loaded onto a monoS 10/100 GL column (Cytiva life sciences) equilibrated in buffer A. The ubiquitin species retained by the column were eluted by running a gradient from 0 to 100% buffer B (50 mM ammonium acetate pH 4.5 and 600 mM NaCl) over 300 ml.

### Isothermal titration calorimetry binding experiments and data analysis

For ITC experiments involving K63Ub_2_, distal ubiquitins contained K48R/K63R mutations and proximal ubiquitins contained D77 mutations to control the polyubiquitin chain length, as described above. For the monoubiquitin-binding experiment in Figure 2*A*, K48R/K63R ubiquitin was used. Before each ITC experiment, the proteins were dialyzed overnight in a solution of 20 mM Hepes pH 7.5, 150 mM NaCl, and 200 μM TCEP. Using a MicroCal iTC_200_ instrument (Malvern), titrations were conducted using a series of 2-μl injections each lasting 4 s, with a minimum of 2 min between injections. Fitting was performed using Origin 7 SR4 (OriginLab). Many of the relatively weak binding interactions produced nonsigmoidal isotherms in which the N values could not be accurately determined. Therefore, we in addition provide the range of *K*_d_ values when fixing N = 1. The N value was set to one in these calculations because the sigmoidal isotherms from the higher affinity binding interactions with the ASCC2 CUE domain and diubiquitin consistently produced N values of approximately one, reflecting the one ASCC2 CUE domain per one K63Ub_2_ binding ratio. The N values were fixed by altering the active concentrations in the cell and syringe during fitting. The first *K*_d_ value in the range corresponds to varying the active concentration in the cell, and the second *K*_d_ value in the range corresponds to varying the active concentration in the syringe.

### Chemical shift mapping experiments

^15^N-labeled ASCC2(465–521) and K63-linked diubiquitin were made following similar expression and purification protocols to those described above, however, after reaching an A_600_ between 0.5 and 0.8, the 1-l aliquots of cells were pelleted, washed with M9 salts, and resuspended in one-third the original volume of minimal media containing ^15^N-labeled ammonium chloride. The resuspended cells recovered for 1 h at 37 °C with 225 rpm shaking before proceeding with the induction of protein expression, as described above. Performing the ^15^N-labeling procedure with either K48R/K63R ubiquitin or D77 ubiquitin and then conjugating the ^15^N-labeled ubiquitin species to the unlabeled ubiquitin species allowed for the production of K63Ub_2_ with the ^15^N-labeled ubiquitin at either the distal or the proximal position, respectively.

^1^H,^15^N-HSQC spectra of ASCC2(465–521) and ubiquitin were recorded using a 600 MHz AVANCE II NMR system at the Biomolecular NMR Center at Johns Hopkins University. Resonances in the ^1^H,^15^N-HSQC spectra of ubiquitin were assigned based on data from Dr Carlos Castañeda (personal communication). Resonance assignments for the ASCC2(465–521) ^1^H,^15^N-HSQC spectra were obtained from 3D ^15^N-edited ^1^H-^1^H NOESY-HSQC and ^15^N-edited ^1^H-^1^H TOCSY-HSQC spectra. NMR data were processed using nmrPipe software (22), and CSP values were measured using the formula $\;d=\sqrt{\delta_{H}^{2}+{\left(0.15\delta_{N}\right)}^{2}}$ by the program CcpNmr Analysis (23) on the NMRBox platform (24). The CSP values in Figure 3 were measured for the titration of 100 μM K63Ub_2,_^15^N-labeled on the distal ubiquitin in Figure 3*A* and ^15^N-labeled on the proximal ubiquitin in Figure 3*B*, with 30 μM, 100 μM, 200 μM, and 500 μM ASCC2(465–521) at 20 °C. The CSP values in Figure 5*A* were measured for 20 μM ^15^N-labeled ASCC2(465–521) alone and in the presence of 681 μM K48R/K63R ubiquitin at 40 °C in a buffer consisting of 20 mM Tris pH 7.0, 100 mM NaCl, and 200 μM TCEP. The CSP values in Figure 5*B* were measured for the titration of 20 μM ^15^N-labeled ASCC2(465–521) with 6 μM, 20 μM, and 40 μM K63Ub_2_ at 40 °C in a buffer consisting of 20 mM Tris pH 7.0, 100 mM NaCl, and 200 μM TCEP. In Figures 3 and 5, resonances that disappeared during the course of the titration are marked by a black bar and assigned values of 0.35 or 0.30 ppm, respectively. NMR data, chemical shift assignments, and CSP values for ^15^N-labeled ASCC2 CUE domain titrated with monoubiquitin and K63Ub_2_ have been deposited to the Biological Magnetic Resonance Data Bank (25) as entries 51,130 and 51,139, respectively. NMR data, chemical shift assignments, and CSP values for ^15^N-labeled K63Ub_2_ titrated with the ASCC2 CUE domain have been deposited to the Biological Magnetic Resonance Data Bank (25) as entries 51,145 (^5^N-labeled on the proximal ubiquitin) and 51,146 (^15^N-labeled on the distal ubiquitin).

### Determining ASCC2 CUE domain binding affinity for monoubiquitin using CSP data

The program CcpNmr Analysis (23) used information from the titration of ^15^N-labeled ASCC2(465–521) with monoubiquitin, as described in the previous section, to determine the *K*_d_ value for ASCC2(465–521) binding monoubiquitin using the formula

$y=A(B+x-\sqrt{{\left(B+x\right)}^{2}-4x)}$ where *y* = δ_obs_, *A* = δ_∞_/2, *B* = 1 + *K*_d_/*a*, *a* = [ASCC2]_tot_, *b* = [Ub]_tot_, and *x* = *b*/*a*. The *K*_d_ value of 39.6 μM ± 1.6 μM reported in Fig. S1 is the average of the *K*_d_ values determined for residues L478, L479, Q500, and L506. These four residues have the largest CSP values recorded for the titration of ^15^N-labeled ASCC2(465–521) with monoubiquitin and are all predicted to be at the ASCC2:ubiquitin-binding interface.

### Modeling the ASCC2 CUE:K63Ub_2_ complex using PyMOL and the HADDOCK server

The PyMOL molecular visualization system (26) and the HADDOCK protein-docking server (16, 17) were used to model the interaction between the ASCC2 CUE domain and K63Ub_2_. First, the interaction between the ASCC2 CUE domain and the distal ubiquitin was modeled based on the solution structure of the gp78 CUE:monoubiquitin complex (PDB ID: 2LVO) (10). PyMOL was used to superimpose the solution structure of the ASCC2 CUE domain (PDB ID: 2DI0) with the structure of the gp78 CUE domain in the ubiquitin-bound complex. The HADDOCK server was then used to model the interaction between the proximal ubiquitin and the ASCC2 CUE domain. To guide the docking experiment, proximal ubiquitin residues with CSP values greater than 2σ, or resonances that disappeared during the NMR titration, were identified as “active” residues. For the ASCC2 CUE domain, residues outside of the conserved hydrophobic patch that decrease the ubiquitin-binding affinity when mutated were identified as active. For the reported model, the active proximal ubiquitin residues were A46, G47, K48, Q49, E64, T66, and L71, and the active ASCC2 CUE domain residues were E467 and S470. In addition, a distance restraint of 1.32 Å between the carbonyl carbon of G76 of the distal ubiquitin and the ε-amino group of K63 of the proximal ubiquitin was used to approximate the isopeptide linkage in the diubiquitin chain. The reported model is the best structure from the highest scoring cluster.

### Immunofluorescence analysis of HA-tagged ASCC2

All immunofluorescence analysis was performed in U2OS cells using WT and mutant forms of ASCC2, expressed in the pHAGE-CMV-3xHA lentivirus (7). Three days after transduction, the cells were treated with 500 μM MMS in complete Dulbecco’s modified Eagle’s medium (DMEM) media for 6 h. U2OS cells were washed once with ice-cold PBS, then extracted with 1× PBS containing 0.2% Triton X-100 and protease inhibitors (Pierce) for 10 to 20 min on ice before fixation with 3.2% paraformaldehyde. The cells were then washed extensively with immunofluorescence wash buffer (1× PBS, 0.5% NP-40, and 0.02% NaN_3_), then blocked with immunofluorescence blocking buffer (immunofluorescence wash buffer plus 10% FBS) for at least 30 min. Primary antibodies were diluted in immunofluorescence blocking buffer overnight at 4 °C. After staining with secondary antibodies (conjugated with Alexa Fluor 488 or 594; Millipore) and Hoechst 33342 (Sigma-Aldrich), where indicated, samples were mounted using Prolong Gold mounting medium (Invitrogen). Epifluorescence microscopy was performed on an Olympus fluorescence microscope (BX-53) using an ApoN 60×/1.49 numerical aperture oil immersion lens or an UPlanS-Apo 100×/1.4 numerical aperture oil immersion lens and cellSens Dimension software. Raw images were exported into Adobe Photoshop, and for any adjustments in image contrast or brightness, the levels function was applied. For foci quantification, at least 100 cells were analyzed in triplicate.

## Data availability

NMR data reported in this article have been deposited in the Biological Magnetic Resonance Data Bank as entries 51,130 (^15^N-labeled ASCC2 CUE domain interacting with monoubiquitin), 51,139 (^15^N-labeled ASCC2 CUE domain interacting with K63Ub_2_), 51,145 (K63Ub_2_^15^N-labeled on the proximal ubiquitin interacting with the ASCC2 CUE domain), and 51,146 (K63Ub_2_^15^N-labeled on the distal ubiquitin interacting with the ASCC2 CUE domain).

## Supporting information

This article contains supporting information.

## Conflict of interest

The authors declare that they have no conflicts of interest with the contents of this article.
